# Prediction of the COVID-19 transmission: a case study of Pakistan

**DOI:** 10.1017/S0950268823000730

**Published:** 2023-05-19

**Authors:** Qurat Ul An Sabir, Ambreen Shafqat, Muhammad Aslam

**Affiliations:** 1 Department of Mathematics, University of Arizona, Tucson, AZ, USA; 2 Department of Urology, Roswell Park Cancer Research Institute, Buffalo, NY, USA; 3 Department of Statistics, Faculty of Science, King Abdulaziz University, Jeddah, Saudi Arabia

**Keywords:** Akaike information criteria, COVID-19, National Command and Operation Center, negative binomial regression model, Poisson regression model, SIRD Model

## Abstract

The world has suffered a lot from COVID-19 and is still on the verge of a new outbreak. The infected regions of coronavirus have been classified into four categories: SIRD model, (1) suspected, (2) infected, (3) recovered, and (4) deaths, where the COVID-19 transmission is evaluated using a stochastic model. A study in Pakistan modeled COVID-19 data using stochastic models like PRM and NBR. The findings were evaluated based on these models, as the country faces its third wave of the virus. Our study predicts COVID-19 casualties in Pakistan using a count data model. We’ve used a Poisson process, SIRD-type framework, and a stochastic model to find the solution. We took data from NCOC (National Command and Operation Center) website to choose the best prediction model based on all provinces of Pakistan, On the values of log L and AIC criteria. The best model among PRM and NBR is NBR because when over-dispersion happens; NBR is the best model for modelling the total suspected, infected, and recovered COVID-19 occurrences in Pakistan as it has the maximum log L and smallest AIC of the other count regression model. It was also observed that the active and critical cases positively and significantly affect COVID-19-related deaths in Pakistan using the NBR model.

## Introduction

Statistical techniques based on the Poisson distribution were used to evaluate all of the cases discussed. The Poisson distribution is a probabilistic distribution that has been used to generate different flexible continuous distributions by compounding methods for modelling survival data relates to counting events. Various overviews based on the Poisson distribution are examined in recent years. For example, Minka et al. [1] introduced the Conway–Maxwell–Poisson (COM-Poisson) distribution for overdispersion and under-dispersion data, in [2] authors also used the COM-Poisson distribution for the fitting discrete data set, the two-parameter distribution called exponential Poisson distribution [3] used for investigating the convergence of the proposed EM scheme, Weibull Poisson distribution studied [4], and Exponentiated Burr XII Poisson distribution for lifetime data set introduced in [5].

Further, the regression model is used to model the relationship between the predictor (*X*) and response (*Y*) variables [6, 7]. The regression model is involved in a linear regression model that is normally distributed and a nonlinear regression model that is not normally distributed is employed [8]. The GLM has involved three components: random component, systematic component, and link function [9]. One of the most useable nonlinear regressions modelled with GLM for lifetime data is Poisson regression. The GLM model was developed to model the relationship between the response and predictor variables and implies that the response variable can be assumed to have the Poisson, binomial, exponential, Gamma, and negative binomial distribution [10]. In Poisson regression, the response variable is considered to have a Poisson distribution. Count information, on the other hand, frequently shows over-dispersion in real-world applications that can be calculated with compound Poisson models (CPM). When the variance is considerably higher than the average, then over-dispersion arises. The information is shown to have over-dispersed when this occurs. CPM can lead to an underestimation of standard errors, resulting in an incorrect conclusion. The aim of the simulation study was how well Poisson and negative binomial regression (NBR) performed during data analysis without any over-dispersion and even some data with over-dispersion. Poisson regression is most suitable for data without any over-dispersion, according to the findings.

In recent years, a lot of authors used CPM, Poisson regression models (PRMs), and compound Poisson processes (CPP) for various diseases such as a study of the major infection diseases human immunodeficiency virus (HIV) described in sub-Saharan Africa using PRM [11], to analyse the risk levels of mortality and deadly illness among MERS-CoV patients in the Middle East during 2012 to 2015, Poisson regression with strong variation and a bootstrap-based expectation maximisation model were utilised to cope with significant incomplete information [12], an interpretation of infinite divisible distribution using Levy–Khintchine Formula with different situations described under the modern interpretations of discrete compound Poisson distributions [13] and a study on the German Children’s cancer database described using CPP monitoring all children malignancies [14]. For further understanding related to CPP, PR, NBR, and generalised Poisson regression, refer to the following for more information [13, 15, 16]. In 2020, a study elaborated on the period of emergence of the infectious molecular releasing characteristic of SARS-coronavirus-infected people that resembled that of flu patients and contrasts with that of SARS-coronavirus-infected patients [17]. The Model of Poisson autoregression of the daily new observed cases dynamically adapt its estimates to explain the evolution of contagion in short and long-term dependence on COVID-19 case counts [18].

Moreover, COVID-19 infection is a transferrable disease that is caused by the severe acute breathing disease known as coronavirus and is now a global pandemic that has affected close to millions of people from different countries. According to the World Health Organisation’s statistics as of March 30, 2020, [19], the mortality rate of persons who had been diagnosed with cases was an average of 4.6% with ranges from 0.2% to a higher level of 15% depending on the age group, which also depends on the health status of the predisposed person. Some of the studies are available on the COVID-19 under various statistical models, for example, a compound Poisson generalised linear model (CPGLM) illustrated [20] with Monte Carlo and random forest models to examine the effect of meteorological elements on COVID-19 in Bangladesh and an SEIR design introduced with different purposed parameters have been overstated to compensate for global spread, maturity reliability spread and death rates [21]. The COVID-19 pandemic in China had accumulated a large data set on the ecology factors of this emerging outbreak, which could be relevant if the epidemic spreads [22] and other studies related to COVID-19 with Pakistani epidemic situation with different models described in [23–25].

COVID-19 has affected whole the world especially in European and African countries, some of the authors described the COVID-19 situations according to their own countries with different statistical techniques such as a study [26] evaluated the metrological factors that influenced the overall coronavirus report as per the generalised Poisson regression in Pakistan, but this study includes the negative impact in Pakistan (202,955 positive cases and 4,118 fatalities) under the CPP and NBR models, a CP model [27] with Poisson subordinator whose random period was an independent Poisson process examined with both exponential and ordinary leaps the first-crossing time case, and a heterogeneous bivariate Weibull regression approach on a CPP of random scale is proposed in [28] for the checking of the several conditions including survivability rate in hereditary hygienic, twins’ birth, and client dentures. It is [29] revealed COVID-19 as a zoonotic virus, related to SARS-CoV and MERS-CoV coronaviruses, and investigated the COVID-19 retroviruses initial reproductive rate (R0) employing various stochastic methods.

The selection of a discrete-time count regression model is made with the aim of modelling and predicting the behaviour of COVID-19 across Pakistan and around the world in the future. These models provide key information, exponential growth rate, and the doubling time of the pandemic and provide a flexible modelling approach for non-negative integers [30, 31]. The PRM and a NBR model are from the generalised linear regression family which is widely used for epidemiological studies. This is strongly believed that the models we presented here are beneficial to be able to track the future trend of diseases like COVID-19 and so forth.

## Research design and methodology

### COVID-19 proposed framework

**Figure 1.**
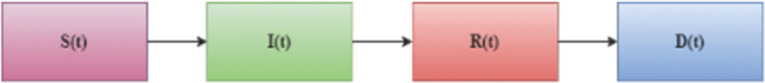
Proposed framework of COVID-19 disease.

We should know that how COVID-19 diseases change from one stochastic state to another during the infection period, as discussed in the COVID-19 Conceptual framework. The parameters taken for analysis are Suspected (S(*t*)), Infected (I(*t*)), Recovered (R(*t*)), and Deaths (D(*t*)) are the four major phases found in a group [34]. A susceptible person who comes into interaction with an infectious individual, as per this theory, is at risk of being infected. An individual who has been infected can either recover or die in case of the virus. So, the total number of (S(*t*)), (I(*t*)), ((R(*t*)), and (D(*t*)) is defined to be constant in this model. Moreover, it is considered anyone who is infected with the virus got infected instantly, without any latent period among stimulation and infection. There seems to be no compensation for the impacts of isolation or quarantine. The period throughout the research is indicated by the ‘*t*’. A proposed framework is used to represent the phases, shown in Figure 1.

The Pakistan Ministry of Health has started to release a special bulletin about COVID-19 infections in Pakistan on 26 February 2020. Data will be taken from the Ministry of Health website. We will use the regression model in Poisson distribution to predict the spread of COVID-19 cases in Pakistan by using data from the beginning until 31 January 2021.

## Methodology and background

### Poisson regression model

The PRM, which is a nonlinear regression analysis of the Poisson distribution, is often used to estimate a response variable with discrete or count data given several explanatory factors. Poisson regression is said to obtain over-dispersion if the variance value is higher than the mean value. Overdispersion has the same effects as if such data types appeared over-dispersion however Poisson regression was also applied, regression coefficients of model parameters may ensure consistency and although inefficient. This is mostly applied when solving issues where the result of a stochastic function can only take count numbers. The Poisson distribution is one of the distributions that fulfil this constraint. It belongs to the exponential distribution family. Assume that ‘*B*’ is the r.v (Coronavirus death ratio) and that the case findings are an event. The ‘*B*’ variable approaches a Poisson distribution [31] with parameter omega *ω* > 0
(1)

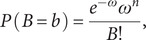

where *n* = 1, 2, 3 is the number of occurrences of an event and ‘*ω*’ is defined as *ω* = Exp[*B*].

The generalised linear model (GLM) can be stated as
(2)

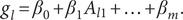

The first function explains how the mean, 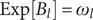 which depends on the linear predictor 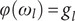 while the second function explains how the variance, 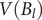 depends on the mean 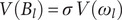 . Where parameter ‘ 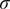 ’ is a constant, assuming 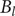 is a Poisson distribution, so

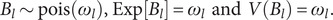

The variance function is defined as follows:


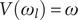
 Ranges from (0, ∞).

A log function is given as

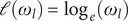



Based on the general linear model (GLM), the linear regression must be associated with the dependent variables with a link variable, which is also the link variable between the linear regression in a matrix form and the Stochastic regression line in this case. Assuming a linear model is as follows:
(3)

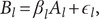

where ‘*A*’ is n* (*m* + 1) vectors of predictors, a column of 1’s *β* is a (*m* + 1) by 1 vector of uncertain parameter, as well as ϵ is a (n* 1) vectors of error values with
(4)

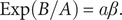



Consider the general linear regression, the linked value and its transportation ‘*B*’ are as below:
(5)

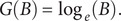

Hence, it may be expressed as
(6)

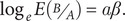



So that, regarding a Poisson regression with the function ‘*β*’ and ‘*a*’ is its input vector, an expected average of the aligned Poisson distribution is taken as
(7)

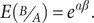

If ‘ 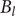 ’ is a response variable with the predictor variable 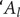 ’, the parameter ‘*β*’ may be evaluated by MLE method. Mathematical models may be used to evaluate the system indicated in equation (7), which is obtained the conditional expectation of response and predictor variables by adopting the logarithm transform of the conditional expectation. Moreover, PRMs’ MLE of the probabilistic area is usually concave, Gradients-based techniques are used as appropriate estimation procedures.

Let ‘ 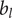 ’ be a random variable and has a positive integer like 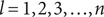 where ‘*n*’ denotes the total number of observations. Hence 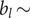 Pois dist. [31]. So, the Probability Mass Function (PMF) will be
(8)

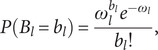



(9)

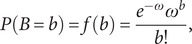

where 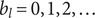 with mean and variance are given as

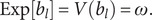



In equation (4), the estimated average of a Poisson Process is defined as

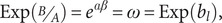

whereas the values of the independent factor 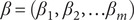 be *k* – dimension vectors of logistic coefficients are unknown, while the average of the estimated stochastic process is presented as 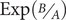 and 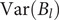 is given as 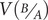 .

The following three factors are indicated by using Poisson regression, which is designed to fit such a rate of fatalities in Pakistan:

*A*_1_ *=* Suspected cases

*A*_2_ *=* Infected cases

*A*_3_ *=* Recovered cases.
(10)

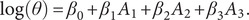

The parameter ‘*β*’ shows the average variation in the independent factor ‘ 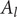 ’ in the logarithmic equation (8) that may be evaluated using the maximum likelihood estimator (MLE) approach.

### Negative binomial regression

To cope with over-dispersion in count data, NBR is being used. As populations are usually varied, specifically distinct to the assumption involved in commonly use basic stochastic methods, over-dispersion is a usual feature of the real statistical approach. In this research, the NBR model [32] is stated as follows:

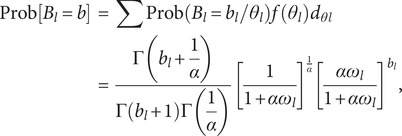



(11)

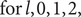

where mean is given as

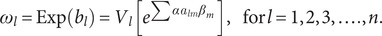



While the variance of 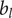 is given as

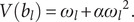



If 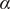 > 0, the method might be called a dispersion parameter. The PRM may be treated with a limited form of the NBR model with 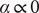 . The MLE statistic is written as
(12)

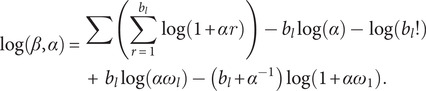

A partially differential of the MLE w.r.t (*β*, *α*) can be used to evaluate the parameter (*β*, *α*). The NBR may not involve the mean = variance but it controls over-dispersion, which happens whenever the value of Variance is larger than the mean value.

The objective is to determine a model with the lowest number of parameters that minimises the Negative likelihood, as shown in the equation below:
(13)

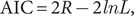

in which ‘*L*’ denotes the likelihood ratio and ‘*R*’ is the number of model parameters.

## Results and discussion

### Finding and discussion

The methods used in this analysis are PRM and NBR which comply mostly with the study’s particular target. The count variables are the total number of fatalities that are the response data, also with events of total suspected, infected, and recovered COVID-19 occurrences as the predictor variables. Continuous count data is used for all the variables.

### Application

From 26 February 2020 to 1 January 2021 secondary information is attained from the National Command and Operation Center (NCOC) website for total suspected, infected, recovered, and deaths COVID-19 occurrences.

A set of count methods, like the PRM and the NBR model have been used to estimate the total number of COVID-19 suspected, infected, recovered, and deaths cases in Pakistan. The data set is taken from 26 February 2020 to 1 January 2021.

Table 1 shows that mean and standard deviation at Mean = 5,166.33 and SD = 4,888.679 for each suspected because of coronavirus. Same as the mean and standard deviation of infected occurrences of coronavirus infection is Mean = 57,580.18 and SD = 60,088.140. The standard deviation mean and of the recovered coronavirus occurrences in the study reveals that Mean = 51,266.59 and SD = 55,753.951. Thus, the mean and standard deviation of the death occurrences of coronavirus is Mean = 1,233.06 and SD = 1,223.407. According to the findings thus far away, the large mean estimate of Covid-19 infected and recovered cases in Pakistan throughout the situation under analysis has resulted in comparatively large cases of coronavirus-associated fatalities.

**Table 1.** Summary statistics of COVID-19 cases in Pakistan

Descriptive statistic				
	Mean	SD	Minimum	Maximum
Suspected	5,166.33	4,888.679	0	21,515
Infected	57,580.18	60,088.140	0	247,249
Recoveries	51,266.59	55,753.951	0	224,664
Deaths	1,233.06	1,223.407	0	4,747

**Figure 2.**
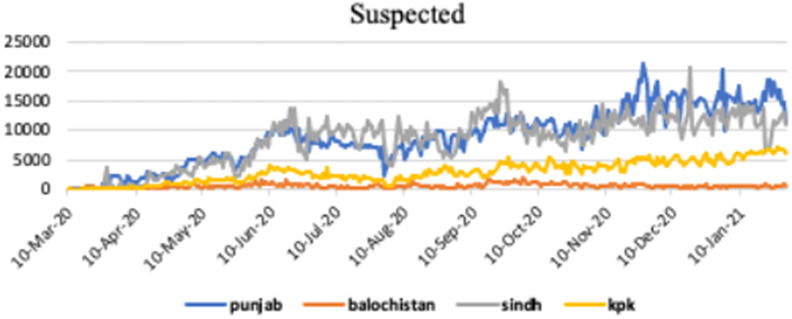
Suspected cases in Punjab, Baluchistan, Sindh and KPK.

Figure 2 shows the graphical representation of suspected cases of COVID-19 infection in different provinces of Pakistan, such as, Punjab, Baluchistan, Sindh and KPK.

**Figure 3.**
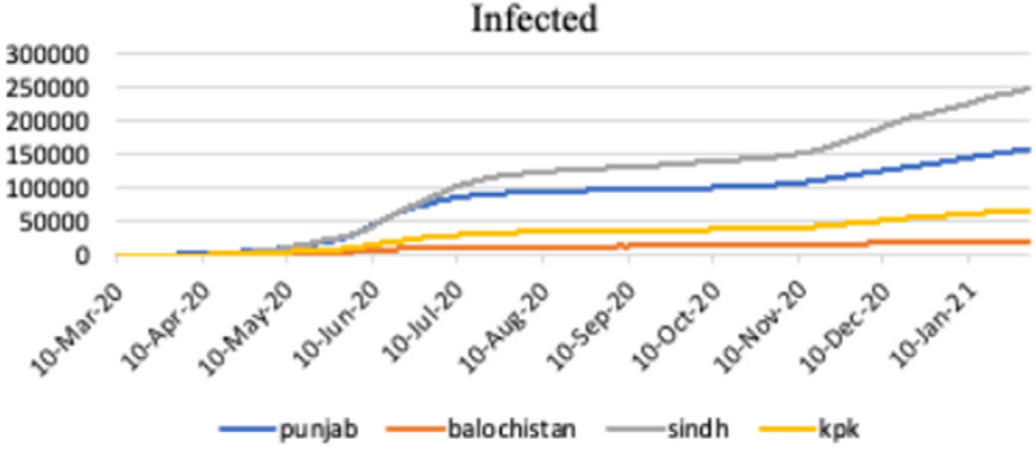
Infected cases in Punjab, Baluchistan, Sindh and KPK.

Figure 3 shows the graphical representation of infected cases of COVID-19 infection in different provinces of Pakistan, such as, Punjab, Baluchistan, Sindh and KPK.

**Figure 4.**
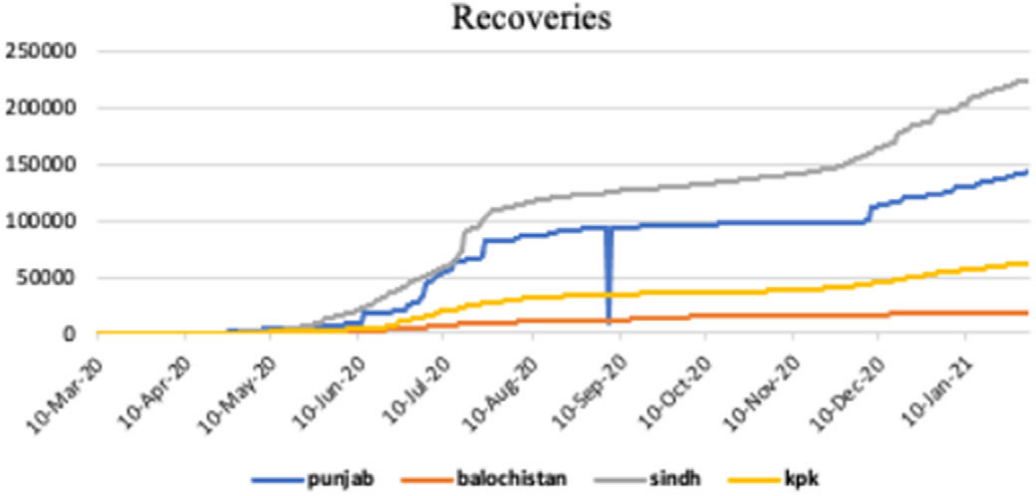
Recoveries in Punjab, Baluchistan, Sindh and KPK.

Figure 4 shows the graphical representation of recovered cases of COVID-19 infection in different provinces of Pakistan, such as, Punjab, Baluchistan, Sindh and KPK.

**Figure 5.**
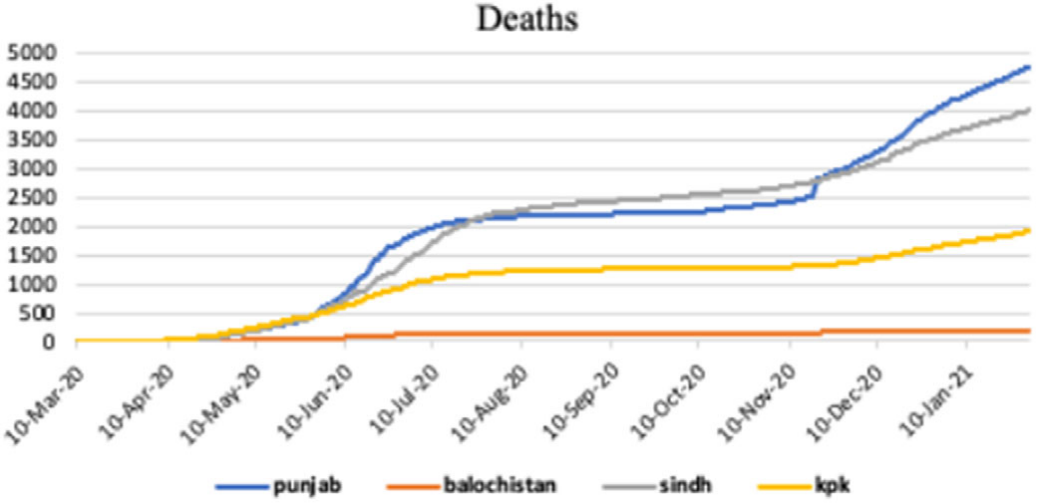
Deaths in Punjab, Baluchistan, Sindh and KPK.

Figure 5 shows the graphical representation of death cases of COVID-19 infection in different provinces of Pakistan, such as, Punjab, Baluchistan, Sindh and KPK.

The omnibus test shows the actual model to the null model that used a test of likelihood-ratio chi-square. The present framework overcomes the null hypothesis since its *p*-value is less than 0.05. It is a likelihood ratio test to perceive all the explanatory variables (suspected, infected, and recovered COVID-19 cases) are significantly outperforming the intercept-only analysis.

Table 2 shows the explanatory variables (i.e., suspected, infected, and recovered cases) have 0.000 *p*-value (*p* < 0.05), in Poisson regression (PR) and NBR models, which indicate an overall statistically significant model.

**Table 2.** Omnibus test of COVID-19 cases in Pakistan

Omnibus test			
Models	Likelihood ratio chi-square	*df*	Significance
Poisson regression	1,198,004.616	3	0.010
Negative binomial regression	1,300.107	3	0.030

The condition of equi-dispersion in Poisson regression analysis, the mean should be equal to the variance. Therefore, the claim is hardly observed, over-dispersion is frequent in many situations. Though over-dispersion is perhaps the most common issue, it is really essential to examine this number firstly. The deviance for all of the models is near to ‘1.2’, which is admissible. Whenever the amount of deviance is reduced by the degree of freedom (*df*), it should be larger than ‘1.2’ to identify over-dispersion; meanwhile, under-dispersion occurs whenever the amount of deviance is lowered with *df* is smaller than ‘1.2’.

Table 3 shows the deviance/*df* and Pearson chi-square/*df* are both more than ‘1.2’, implying that the numbers of COVID-19 occurrences in Pakistan are over-dispersed, based on the PRM. The analysis is performed by NBR to get the better solution as this approach may handle the dispersion parameter, and the resulting log-likelihood and Akaike informatics criteria (AIC) results are (−209,814.870 and 419,637.741) respectively.

**Table 3.** Association of variables with model selection (PRM)

Goodness-of -fit			
	Value	*df*	Value/*df*
Deviance	409,680.895	1,261	324.886
Pearson chi-square	362,033.002	1,261	287.100
Log–likelihood	−209,814.870	1,261	−166.388
Akaike’s information criterion (AIC)	419,637.741	1,261	332.782

Table 4 indicates both amounts of Pearson chi-square/*df* and deviance/*df* are below ‘1.2’ (i.e., 1.069 and 0.687, respectively), implying that the amount of COVID-19 occurrences in Pakistan are under-dispersion, and have log-likelihood and −9,664.914 and 19,337.828, respectively.

**Table 4.** Association of variables with model selection (NBR)

Goodness-of-fit			
	Value	*df*	Value/*df*
Deviance	1,473.910	1,261	1.069
Pearson chi-square	866.075	1,261	0.687
Log-likelihood	−9,664.914	1,261	−7.664
Akaike’s information criterion	19,337.828	1,261	15.335

The reported COVID-19 death cases are a count, and then this form of data seems to have a Poisson distribution. To analyse the variables that impact the number of COVID-19 cases of deaths, the Poisson regression modelling is used. The maximum likelihood estimator (MLE) technique is commonly used to predict the parameters of Poisson regression.

Table 5 shows *p*-values for all parameters that are smaller than 0.05, indicating that the parameters 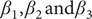 have a significant impact on results. Consequently, the PRM is given as

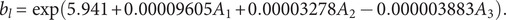

Explanatory factors that determine the coronavirus fatalities in Pakistan are the coronavirus suspected ( 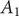 ), infected ( 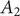 ) and recovered 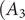 ) occurrences.

**Table 5.** Multiple terms test between diagnose disease and respondence in Pakistan (PRM)

Parameter estimates (PRM)							
Parameter	*B*	SE	95% wald confidence interval		Hypothesis test		
			Lower	Upper	Wald chi-square	*df*	Significance
(Intercept)	5.941	0.0017	5.938	5.944	11,706,409.09	1	0.000
Suspected	9.605E-5	2.3205E-7	8.559E-5	8.650E-5	137,502.939	1	0.040
Infected	3.278E-5	8.7030E-8	3.108E-6	3.449E-6	1,419.038	1	0.010
Recoveries	−3.883E-6	8.9952E-8	3.707E-6	4.059E-6	1,863.456	1	0.30
(Scale)	1						

The NBR is often used to predict count observation as well as obtain dispersion measurement to tackle the limitation of over-dispersion related to the PRM. Consequently, the NBR model is given as

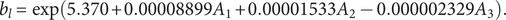


Table 6 reveals that basically, factors are statistically significant at 5% level, also *p* < 0.05. With exception of the recovered cases, the NBR model shows that two of the COVID-19 factors are positive. This indicates that when using the NBR model to estimate the parameter, a 1% rise in suspected and infected COVID-19 instances might result in a 0.009% and 0.002% higher in COVID-19-related deaths in Pakistan. COVID-19 patients that are suspected and infected have a positive effect on the deaths related to coronavirus in Pakistan. Consequently, at the 5% level, the recovered occurrences have a negative value (−0.000002329), which implies a 1% increase in recovered coronavirus occurrences in Pakistan throughout the time period under consideration may occur in a −0.0002% reduction in the number of deaths caused by infectious disease in Pakistan and outcome often reveals that recovered occurrences may have an adverse impact of the coronavirus fatalities.

**Table 6.** Multiple terms test between diagnose disease and respondence in Pakistan (NBR)

Parameter estimates (NBR)							
Parameter	*B*	SE	95% wald confidence interval		Hypothesis test		
			Lower	Upper	Wald chi-square	*df*	Significance
(Intercept)	5.370	0.0489	5.274	5.466	12,034.059	1	0.030
Suspected	8.899E-5	1.3963E-5	6.162E-5	0.000	40.617	1	0.010
Infected	1.533E-5	3.5393E-6	8.391E-6	2.226E-5	18.756	1	0.000
Recoveries	−2.329E-6	3.2563E-6	−8.712E-6	4.053E-6	0.512	1	0.050
(Scale)	1						
(Negative Binomial)	1

**Table 7.** Selected count data model for COVID-19 cases

Provinces	Models			
	Poisson regression		Negative binomial regression	
	AIC	Log-likelihood	AIC	Log-likelihood
Punjab	39,984.215	−19,988.107	5,244.866	−2,618.433
Balochistan	4,018.361	−2,005.180	3,491.162	−1,741.581
Sindh	79,922.877	−39,957.439	5,252.483	−2,622.242
KPK	38,215.096	−19,103.548	4,971.439	−2,481.719
Overall	419,637.741	−209,814.870	19,337.828	−9,664.914

Table 7 shows the association of variables with a model selection of the two counts data, specifically PRM and NBR. The AIC is often used to evaluate approaches. AIC is a way to estimate the likelihood of a model that can forecast results depending on in-sample fits. As compared to another method, a model performs best if its AIC is the lowest.

**Table 8.** Descriptive statistics of COVID-19 (female) cases in Pakistan

Descriptive statistics				
	Mean	SD	Minimum	Maximum
Suspected	1,446.59	1,368.817	0	6,024
Infected	16,122.44	16,824.680	0	69,230
Recoveries	13,840.42	15,559.324	0	62,906
Deaths	345.26	342.552	0	1,329

The NBR has the smallest AIC and likelihood values, as stated by the consequence: (5,244.866, −2,618.433) in Punjab, (3,491.162, −1,741.581) in Baluchistan, (5,252.483, −2,622.242) in Sindh, (4,971.439, −2,481.719) in KPK and (19,337.828, −9,664.914) in Pakistan, and is a selected count model for modelling suspected, infected and recovered coronavirus epidemic occurrences in all Provinces of Pakistan.

### Comparison of COVID-19 cases between male and female

The study analysed the effect of the COVID-19 death rates on gender. However, females have a better immune system as compared to males. So, male occurrences are more severe than female occurrences. Males with COVID-19 are at a higher risk for adverse outcomes and deaths. The statistical analysis of the COVID-19 data set shows that men suffer a significantly higher fatality rate than women.

Table 8 shows the mean and standard deviation of females at (Mean = 1,446.59 and SD = 1,368.817) for each suspected because of COVID-19. Same as the mean and standard-deviation of infected occurrences of coronavirus (Mean = 16,122.44 and SD = 16,824.680). The mean and standard deviation of the recovered occurrences of coronavirus in the study reveal that Mean = 13,840.42 and SD = 15,559.324. Thus, the mean and standard deviation of the death occurrences of COVID-19 are Mean = 345.26 and SD = 342.552. According to the findings thus far away, the large mean estimate of COVID-19-infected and recovered (female) cases in Pakistan during the period under analysis has resulted in comparatively large cases of COVID-19-associated deaths.

**Table 9.** Omnibus test of COVID-19 (female) cases in Pakistan

Omnibus test			
Models	Likelihood ratio chi-square	*df*	Significance
Poisson regression	357,878.762	3	0.050
Negative binomial regression	1,456.218	3	0.030

Table 9 shows that the explanatory factors (i.e., suspected, infected, and recovered occurrences of coronavirus) have a 0.000 *p*-value (i.e., *p* < 0.05), showing a statistically significant appropriate assessment in PRM and NB).

**Table 10.** Association of variables with model selection (PRM) of COVID-19 (female) cases in Pakistan

Goodness-of-fit			
	Value	*df*	Value/*df*
Deviance	125,351.956	1,308	95.835
Pearson chi-square	110,179.713	1,308	84.235
Log-likelihood	−66,865.403	1,308	−51.120
Akaike’s information criterion (AIC)	133,738.807	1,308	102.247

Table 10 demonstrates that the amount of deviance/*df* and Pearson chi-square/*df* are both more than ‘1.2’, implying that the number of COVID-19 (female) occurrences in Pakistan is over-dispersed, based on PRM. The analysis is performed by NBR to get the better solution as this approach may handle the dispersion parameter, and the resulting log-likelihood and AIC results are −66,865.403 and 133,738.807, respectively.

**Table 11.** Association of variables with model selection (NBR) of COVID-19 (female) cases in Pakistan

Goodness-of-fit			
	Value	*df*	Value/*df*
Deviance	1,747.278	1,308	1.036
Pearson chi-square	1,013.607	1,308	0.775
Log-likelihood	−8,253.522	1,308	−6.310
Akaike’s information criterion (AIC)	16,515.044	1,308	12.626

Table 11 indicates that the amounts of deviance/*df* and Pearson chi-square/*df* are both below ‘1.2’ (i.e., 1.036 and 0.775, respectively), implying that the amount of COVID−19 (female) occurrences in Pakistan are under-dispersion, having log-likelihood and AIC numbers −8,253.522 and 16,515.044, respectively.

**Table 12.** Multiple terms test between diagnose disease and respondence (female) in Pakistan (PRM)

Parameter estimates							
Parameter	*B*	SE	95% wald confidence interval		Hypothesis test		
			Lower	Upper	Wald chi-square	*df*	Significance
(Intercept)	4.615	0.0033	4.608	4.621	2,002,553.706	1	0.000
Suspected	0.0007	1.5632E-6	0.000	0.000	40,519.410	1	0.057
Infected	4.415E-5	5.8732E-7	1.300E-5	1.530E-5	580.499	1	0.046
Recoveries	−1.190E-5	6.0770E-7	1.071E-5	1.309E-5	383.677	1	0.027
(Scale)	1						

Table 12 shows the *p*-value of all parameters are lower than 0.05, indicating the parameters 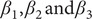 have a significant impact on results. Therefore, the PRM is given as

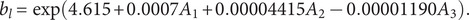

**Table 13.** Multiple terms test between diagnose disease and respondence (female) in Pakistan (NBR)

Parameter estimates (NBR)							
Parameter	*B*	SE	95% wald confidence interval		Hypothesis test		
			Lower	Upper	Wald chi-square	*df*	Significance
(Intercept)	3.997	0.0478	3.903	4.090	6,992.882	1	0.000
Suspected	4.569E-4	5.0417E-5	0.000	0.000	36.575	1	0.050
Infected	1.589E-5	1.2910E-5	4.058E-5	9.119E-5	26.045	1	0.030
Recoveries	−1.538E-5	1.1826E-5	−3.855E-5	7.804E-6	1.690	1	0.020
(Scale)	1						
(Negative binomial)	1						

Explanatory factors which determine the coronavirus fatalities in Pakistan are the coronavirus suspected ( 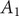 ), infected ( 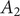 ) and recovered ( 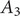 ) occurences.

Consequently, the NBR is given as

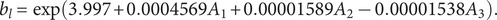



Table 13 reveals that almost all factors are significant statistically at a 0.05 level of significance, and *p*-values of all paramaters are lower than 0.05. Except for recovered cases, the NBR model shows that two of the COVID-19 (female) factors are positive. This indicates that when using the NBR model to estimate the parameter, a 1% rise in suspected and infected COVID-19 (female) instances might result in a 0.05% and 0.002% higher in COVID-19-related deaths in Pakistan. COVID-19 patients that are suspected and infected have a direct positive impact on deaths related to COVID-19 in Pakistan. Consequently, at the 5% level, the recovered occurrences have a negative value (−0.00001538), which implies that a 1% rise in recovered coronavirus occurrences in Pakistan under the period of analysis may occur in a (−0.002%) reduction in deaths caused by COVID-19 in Pakistan, and the outcome often reveals that recovered occurrences may have a negative impact of the COVID-19 (female) deaths.

**Table 14.** Descriptive statistics of COVID-19 (male) cases in Pakistan

Descriptive statistics				
	Mean	SD	Minimum	Maximum
Suspected	3,719.74	3,519.862	0	15,491
Infected	41,457.74	43,263.461	0	178,019
Recoveries	35,589.65	40,009.649	0	161,758
Deaths	887.79	880.855	0	3,418

Table 14 shows that there is a mean and standard deviation of males at Mean = 3,719.74 and SD = 3,519.862 for each suspected because of coronavirus infection. Same as the mean and standard deviation of infected occurrences of coronavirus are Mean = 41,457.74 and SD = 43,263.461. The mean and standard deviation of the recovered occurrences of coronavirus disease upon the study reveals that Mean = 35,589.65 and SD = 40,009.649. Thus, the mean and standard deviation of the death occurrences of coronavirus infection are Mean = 887.79 and SD = 880.855. According to the findings thus far away, the large mean estimate of coronavirus infected and recover (male) occurrences in Pakistan during the period under analysis resulted in a comparatively large amount of COVID-19-associated deaths.

**Table 15.** Omnibus test of COVID-19 (male) cases in Pakistan

Omnibus test			
Models	Likelihood ratio chi-square	*df*	Significance
Poisson regression	920,295.977	3	0.040
Negative binomial regression	1,462.118	3	0.000

Table 15 shows that the explanatory factors (i.e., suspected, infected, and recovered occurrences of coronavirus) have a 0.000 *p*-value (i.e., *p* < 0.05), showing a statistically significant appropriate assessment in PRM and NBR.

**Table 16.** Association of variables with model selection (PRM)

Goodness-of-fit			
	Value	*df*	Value/*df*
Deviance	322,215.894	1,308	246.342
Pearson chi-square	283,300.674	1,308	216.591
Log-likelihood	−165,882.729	1,308	−126.822
Akaike’s information criterion	331,773.459	1,308	253.649

Table 16 demonstrates that amount of Pearson chi-square/*df* and deviance/*df* are both more than ‘1.2’, implying that the number of COVID-19 (male) occurrences in Pakistan is over-dispersed, based on PRM. The analysis is performed by NBR to get a better solution as this approach may handle the dispersion parameter, and the resulting log-likelihood and AIC results are −165,882.729 and 331,773.459, respectively.

**Table 17.** Association of variables with model selection (NBR)

Goodness-of-fit			
	Value	*df*	Value/*df*
Deviance	1,915.229	1,308	1.064
Pearson chi-square	1,023.804	1,308	0.783
Log-likelihood	−9,488.507	1,308	−7.254
Akaike’s information criterion (AIC)	18,985.015	1,308	14.515

Table 17 indicates that amounts of deviance/*df* and Pearson chi-square/*df* are both below ‘1.2’ (i.e., 1.064 and 0.783), respectively, then it is implying that the number of COVID-19 (male) occurrences in Pakistan are under-dispersion, having log-likelihood and AIC numbers are (−9,488.507, and 18,985.015) respectively.

**Table 18.** Multiple terms test between diagnose disease and respondence (male) in Pakistan (PRM)

Parameter estimates (NBR)							
Parameter	*B*	SE	95% wald confidence interval		Hypothesis test		
			Lower	Upper	Wald chi-square	*df*	Significance
(Intercept)	5.559	0.0020	5.555	5.563	7,472,469.618	1	0.000
Suspected	0.00087	3.7909E-7	0.000	0.000	104,183.125	1	0.040
Infected	5.503E-6	1.4244E-7	5.223E-6	5.782E-6	1,492.343	1	0.031
Recoveries	−4.631E−6	1.4738E-7	4.342E-6	4.920E-6	987.251	1	0.037
(Scale)	1						

Table 18 shows the parameters of the *p*-values are lower than 0.05, indicating that the parameters 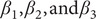 have a significant impact on results. Consequently, the PRM is given as

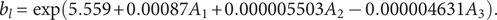

Explanatory factors which determine the coronavirus fatalities in Pakistan are the coronavirus suspected occurrences ( 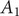 ), infected occurrences ( 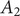 ), and recovered occurrences ( 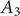 ).

**Table 19.** Multiple terms test between diagnose disease and respondence (male) in Pakistan (NBR)

Parameter estimates (NBR)							
Parameter	*B*	SE	95% wald confidence interval		Hypothesis test		
			Lower	Upper	Wald chi-square	*df*	Significance
(Intercept)	4.937	0.0477	4.843	5.031	10,698.219	1	0.000
Suspected	0.00067	1.9607E-5	8.005E-5	0.000	36.514	1	0.030
Infected	2.579E-5	5.0227E-6	1.595E-5	3.564E-5	26.373	1	0.000
Recoveries	−6.080E-6	4.5988E-6	−1.509E-5	2.934E-6	1.748	1	0.010
(Scale)	1						
(Negative binomial)	1						

Therefore, the NBR is given as

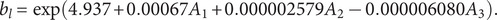



Table 19 reveals that almost all the parameters are significant statistically at the 5% level of significance, and the parameters of the *p*-values are all less than 0.05. Apart from the recovered cases, the NBR model shows that two of the COVID-19 factors are positive. This indicates that when using the NBR model to estimate the parameter, a 1% rise in suspected and infected COVID-19 (male) instances might result in a 0.067% and 0.003% higher in COVID-19 related deaths (male) in Pakistan. COVID-19 patients that are suspected and infected have a direct positive impact on deaths related to COVID-19 in Pakistan. Consequently, at the 5% level, the recovered occurrences have a negative value (−0.000006080) which implies that a 1% rise in recovered COVID-19 occurrences in Pakistan under the period of analysis may occur in a (−0.0006%) reduction in deaths caused by COVID-19 in Pakistan, and the outcome often reveals that recovered occurrences may have a negative impact of the Covid19 (male) deaths.

**Table 20.** Selected count data model for COVID-19 cases between male and female

Genders	Models			
	Poisson regression		Negative binomial regression	
	AIC	Log-likelihood	AIC	Log-likelihood
Male	331,773.459	−165,882.729	18,985.015	−9,488.507
Female	133,738.807	−66,865.403	11,515.044	−6,053.522

Table 20 shows that the NBR has the smallest AIC and log-likelihood values, as stated by the consequence: (18,985.015, −9,488.507) in males, respectively, while (11,515.044, −6,053.522) in females and is the selected count data model for modelling the suspected, infected and recovered Coronavirus widespread occurrences between males and females in Pakistan. Hence the resulted AIC and log-likelihood shows that males are more prone than females.

## Conclusion

We have used two logistic count regression models to predict the third wave of Covid-19. These models proved very beneficial in the prediction of Covid-19. We have fitted two selected count Regression models on suspected, infected, and recovered cases as factors that impact the novel coronavirus disease 2019, (COVID-19) deaths in Pakistan. The competitive results indicate that the proposed reliability-based regression model has higher performance in predicting the deterioration of COVID-19 patients compared to the classic accuracy-based regression model [33]. During the period under analysis, we provide an overview of the (suspected, infected, recovered and deaths) COVID-19 occurrences observed in different provinces of Pakistan from the period of 26 February 2020 to 31 January 2021. The study shows that the PRM analysis is unable to handle over-dispersion, so another method of the PRM (logistic regression) including the NBR is applied in the prediction. It can be determined from the outcomes of the model’s selection process which include log L and AIC. So, the NBR has the lowest value of AIC and greatest value of log L from the two-selection process, it is considered as the better model. The positively significant impact of suspected and infected occurrences over the coronavirus fatality rate in different provinces of Pakistan, such as Punjab, Baluchistan, Sindh and KPK, is a unique result of this study.

For male cases by PRM model: ( 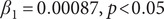 ) a 1% rise in suspected occurrences resulted in a 0.09% of rising in fatality number in Pakistan, whereas ( 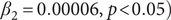 indicates that 1% rise in infected occurrences resulted in a 0.006% of rise in the fatalities in Pakistan. For male cases by NBR model: ( 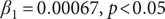 ) a 1% rise in suspected occurrences resulted in a 0.07% of rise in the fatalities in Pakistan whereas ( 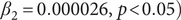 reveals that 1% rise in critical occurrences resulted in a 0.003% of rise fatality’s number in Pakistan.

Female cases by PRM model: ( 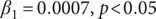 ) indicates that 1% rise in suspected occurrences resulted in 0.07% of rise in several fatalities, whereas ( 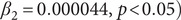 indicates that a 1% rise in infected occurrences resulted in 0.004% of rise in the number of fatalities in Pakistan. Female by NBR model: ( 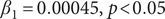 ) indicates that 1% rise in suspected occurrences resulted in 0.05% of rise in the number of fatalities whereas ( 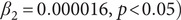 reveals that 1% rise in infected occurrences resulted in a 0.002% of the rise in the number of fatalities in Pakistan.

Whenever there is evidence of over-dispersion in the counts, the NBR is said to be an appropriate model for evaluating the factors that impact the coronavirus fatalities in various provinces. The suggestions based on the current study and results are NBR analysis must be utilised to model the daily suspected, infected, and recovered occurrences as variables affecting coronavirus disease-related deaths and NCOC should be focused more on suspected and infected instances as they are the main factors of COVID-19 related fatalities in Pakistan. This study was done on the data of non-vaccinated people while a similar kind of study can be done on vaccinated people, and we can make a comparison of the efficacy of the vaccine in the future.

The same proposed prediction model may be built separately for different countries with unique infrastructure, atmospheric and demographic conditions attributes, and symptomatic patients. The model can be used by the different organisations that are researching different contagious diseases to be focused more on suspected and infected cases as they are the main factors of COVID-19-related deaths in Pakistan. The study recommended that the Pakistani government, Ministry of Health, and administration should give more awareness and protections for societies, and they should also open more COVID-19 laboratory testing centres. Generally, the obtained results of this study may help Pakistani decision-makers put a short-term future to face this pandemic.

## Data Availability

The data are available to the authors upon a reasonable request.

## References

[1] Minka TP, Shmeuli G, Kadane JB, Borle S and Boatwright P (2003) *Computing with the COM-Poisson Distribution. PA: Department of 776.*

[2] Shmueli G, Minka TP, Kadane JB, Borle S and Boatwright P (2005) A useful distribution for fitting discrete data: Revival of the Conway–Maxwell–Poisson distribution. Journal of the Royal Statistical Society: Series C (Applied Statistics) 54, 127–142.

[3] Kuş C (2007) A new lifetime distribution. Computational Statistics & Data Analysis 51, 4497–4509.

[4] Hemmati F, Khorram E and Rezakhah S (2011) A new three-parameter ageing distribution. Journal of Statistical Planning and Inference 141, 2266–2275.

[5] da Silva VR, Gomes-Silva F, Ramos MWA and Cordeiro GM (2015) The exponentiated Burr XII Poisson distribution with application to lifetime data. International Journal of Statistics and Probability 4(4), 112. 10.5539/ijsp.v4n4p112.

[6] Cameron AC and Trivedi PK (2013) *Regression Analysis of Count Data.*

[7] Scott LJ (1997) Regression models for categorical and limited dependent variables. Advanced Quantitative Techniques in the Social Sciences 7, 328.

[8] Weisberg S (2005) Applied Linear Regression. Hoboken, NJ: John Wiley & Sons.

[9] Agresti A (2003) Categorical Data Analysis. Hoboken, NJ: John Wiley & Sons.

[10] McCullagh P and Nelder JA (1989) Generalized Linear Models, 2nd Edn. New York: Chapman and Hall.

[11] Monday Osagie A (2017) Fitting a Poisson regression model to reported deaths from HIV/AIDS in Nigeria. International Journal of Statistical Distributions and Applications 3, 56.

[12] Rivers CM, Majumder MS and Lofgren ET (2016) Risks of death and severe disease in patients with Middle East respiratory syndrome coronavirus, 2012–2015. American Journal of Epidemiology 184, 460–464. 27608662 10.1093/aje/kww013 PMC5023790

[13] Zhang H and Li B (2016) Characterizations of discrete compound Poisson distributions. Communications in Statistics – Theory and Methods 45, 6789–6802.

[14] Westermeier T and Michaelis J (1995) Applicability of the Poisson distribution to model the data of the German Children’s cancer registry. Radiation and Environmental Biophysics 34, 7–11. 7604164 10.1007/BF01210539

[15] Kruczek P, Polak M, Wyłomańska A, Kawalec W and Zimroz R (2018) Application of compound Poisson process for modelling of ore flow in a belt conveyor system with cyclic loading. International Journal of Mining, Reclamation and Environment 32, 376–391.

[16] Wu Y, Jing W, Liu J, Ma Q, Yuan J, Wang Y, Du M and Liu M (2020) Effects of temperature and humidity on the daily new cases and new deaths of COVID-19 in 166 countries. Science of the Total Environment 729, 139051. 32361460 10.1016/j.scitotenv.2020.139051 PMC7187824

[17] Zou L, Ruan F, Huang M, Liang L, Huang H, Hong Z, Yu J, Kang M, Song Y, Xia J, Guo Q, Song T, He J, Yen HL, Peiris M and Wu J (2020) SARS-CoV-2 viral load in upper respiratory specimens of infected patients. New England Journal of Medicine 382, 1177–1179. 32074444 10.1056/NEJMc2001737 PMC7121626

[18] Agosto A, Campmas A, Giudici P and Renda A (2021) Monitoring COVID‐19 contagion growth. Statistics in Medicine 40, 4150–4160. 33973656 10.1002/sim.9020 PMC8242489

[19] WHO (2020) *Global Surveillance for COVID-19 Disease Caused by Human Infection with the 2019 Novel Coronavirus. Interim Guidance, 27 February 2020.*

[20] Islam ARMT, Hasanuzzaman M, Shammi M, Salam R, Bodrud-Doza M, Rahman MM, Mannan MA and Huq S (2021) Are meteorological factors enhancing COVID-19 transmission in Bangladesh? Novel findings from a compound Poisson generalized linear modeling approach. Environmental Science and Pollution Research 28, 11245–11258. 33118070 10.1007/s11356-020-11273-2 PMC7594949

[21] Ferguson NM, Laydon D and Nedjati-Gilani G (2020) *Impact of Non-Pharmaceutical Interventions (NPIs) to Reduce COVID19 Mortality and Healthcare Demand. Imperial College COVID-19 Response Team.*

[22] Byass P (2020) Eco-epidemiological assessment of the COVID-19 epidemic in China, January–February 2020. Global Health Action 13, 1760490. 32404043 10.1080/16549716.2020.1760490 PMC7269037

[23] Khan F, Saeed A and Ali S (2020) Modelling and forecasting of new cases, deaths and recover cases of COVID-19 by using vector autoregressive model in Pakistan. Chaos, Solitons & Fractals 140, 110189. 32834659 10.1016/j.chaos.2020.110189 PMC7405884

[24] Khan S, Khan M, Maqsood K, Hussain T, Noor-Ul-Huda and Zeeshan M (2020) Is Pakistan prepared for the COVID‐19 epidemic? A questionnaire‐based survey. Journal of Medical Virology 92, 824–832. 32237161 10.1002/jmv.25814 PMC7228297

[25] Hussain A (2020) What Do Confirmed Numbers Tell Us? Using an adapted SEIR model for Estimation of COVID-19 in Pakistan. Islamabad: Pakistan Institute of Development Economics.

[26] Raza A, Khan MTI, Ali Q, Hussain T and Narjis S (2021) Association between meteorological indicators and COVID-19 pandemic in Pakistan. Environmental Science and Pollution Research 28, 40378–40393. 33052566 10.1007/s11356-020-11203-2 PMC7556579

[27] Hanagal DD (2010) Modeling heterogeneity for bivariate survival data by the compound Poisson distribution with random scale. Statistics & Probability Letters 80, 1781–1790.

[28] Di Crescenzo A, Martinucci B and Zacks S (2015) Compound Poisson process with a Poisson subordinator. Journal of Applied Probability 52, 360–374.

[29] Liu Y, Gayle AA, Wilder-Smith A and Rocklöv J (2020) The reproductive number of COVID-19 is higher compared to SARS coronavirus. Journal of Travel Medicine 27, taaa021. 10.1093/jtm/taaa021. 32052846 PMC7074654

[30] Fekedulegn D, Andrew M, Violanti J, Hartley T, Charles L and Burchfiel C (2010) Comparison of statistical approaches to evaluate factors associated with metabolic syndrome. The Journal of Clinical Hypertension 12, 365–373. 20546380 10.1111/j.1751-7176.2010.00264.x PMC8673351

[31] Kianifard F and Gallo PP (1995) Poisson regression analysis in clinical research. Journal of Biopharmaceutical Statistics 5, 115–129. 7613557 10.1080/10543409508835101

[32] Juarez‐Colunga E and Dean CB (2020) Negative binomial regression. In Wiley StatsRef: Statistics Reference Online. Hoboken, NJ: Wiley, pp. 1–8.

[33] Bakhtiarvand N, Khashei M, Mahnam M, and Hajiahmadi S (2022) “A novel reliability-based regression model to analyze and forecast the severity of COVID-19 patients,” BMC Medical Informatics and Decision Making 22(1), 123, doi: 10.1186/s12911-022-01861-2. 35513811 PMC9069125

[34] Nair R, Soni M, Bajpai B, Dhiman G, and Sagayam KM (2022). Predicting the death rate around the world due to COVID-19 using regression analysis. International Journal of Swarm Intelligence Research (IJSIR), 13(2), 1–1

